# Dramatic Response to Ensartinib in Metastatic Neuroendocrine Tumors With a Novel *CEP44‐ALK* Fusion: A Case Report and Literature Review

**DOI:** 10.1111/crj.70040

**Published:** 2024-12-12

**Authors:** Haiyang Chen, Yingxi Wu, Xuan Wu, Kai Wang, Qingxin Xia, Qiming Wang

**Affiliations:** ^1^ Department of Internal Medicine The Affiliated Cancer Hospital of Zhengzhou University & Henan Cancer Hospital Zhengzhou China; ^2^ Academy of Medical Science Zhengzhou University Zhengzhou China; ^3^ Medical Center Geneplus‐Beijing Beijing China; ^4^ Department of Pathology The Affiliated Cancer Hospital of Zhengzhou University & Henan Cancer Hospital Zhengzhou China; ^5^ Institute of Cancer Research Henan Academy of Innovations in Medical Science Zhengzhou China

**Keywords:** case report, ensartinib, neuroendocrine tumor, next‐generation sequencing, novel *CEP44‐ALK* rearrangement

## Abstract

Neuroendocrine tumor (NET) is a deadly malignancy disease that can be found anywhere in the body. The lack of tumor‐specific treatment led to the worse prognosis of NET. Anaplastic lymphoma kinase‐tyrosine kinase inhibitors (ALK‐TKIs), such as alectinib and crizotinib, have been used in the treatment of NET patients with ALK rearrangement. However, the response to ensatinib in NET patients with rare ALK fusion has been rarely reported. Here, we report a 55‐year‐old Chinese female patient with NET (atypical carcinoid tumor) and a novel CEP44‐ALK rearrangement identified by next‐generation sequencing (NGS). NGS can provide more information on mutation landscape for rare neuroendocrine tumors to guide treatment and assist in clinical decisions by presenting molecular changes. The patient received ensartinib (225 mg/day) for 18 months until disease progression in June 2024 and achieved a radiographic partial response. Although patients with ALK fusions showed response to ensatinib in nonsmall cell lung cancer (NSCLC), this study first reports a metastatic NET case with a novel CEP44‐ALK rearrangement that responded favorably to ensartinib.

## Graphical abstract

A 55‐year‐old Chinese female was diagnosed with a neuroendocrine tumor harboring a novel CEP44‐ALK fusion by next‐generation sequencing (NGS)‐based genetic testing. The patient was administered oral ensartinib 225 mg per day and achieved a radiographic partial response after 3 months of treatment. The treatment has lasted 18 months until disease progression in June 2024 and achieved a radiographic partial response.

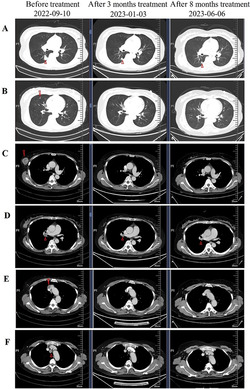

## 1 Introduction

Anaplastic lymphoma kinase (ALK) gene encodes anaplastic lymphoma kinase and activates downstream signaling pathway of cell survival, proliferation, and oncogenesis [1]. More than 90 different fusion partners for ALK were reported, and echinoderm microtubule‐associated protein‐like 4 (EML4) gene is the most common [2]. Patients harboring ALK fusions can benefit from multiple ALK‐tyrosine kinase inhibitors (TKIs), which have significantly improved the prognosis of these patients. As a second‐generation of ALK‐TKI, ensartinib hold superior efficacy to crizotinib and had been approved for the first‐line treatment in patients with ALK‐positive nonsmall cell lung cancer (NSCLC) [3]. In addition, the large‐cell neuroendocrine carcinomas (LCNECs) with ALK fusions also exhibited response to ALK‐TKIs [4]. However, the response to ensartinib in neuroendocrine tumor (NET) with nonclassic ALK rearrangement has rarely been reported. Here, we present the first case of metastatic NET patient with a novel *CEP44‐*ALK rearrangement, who exhibited a long‐term radiographic response to ensartinib.

## 2 Case Presentation

A 55‐year‐old Chinese female came to our hospital in September 2022 with right lower back and abdominal pain for several days. Computed tomography (CT) scan revealed a 24 mm × 13 mm mass in the lower lobe of right lung, multiple nodules in bilateral lung lobes, and a 35 mm × 32 mm mass in the upper outer quadrant of right breast. Additionally, there was evidence of enlarged lymph nodes in the mediastinum, right hilum, supraclavicular region, and chest wall. Magnetic resonance imaging (MRI) and positron‐emission tomography (PET)‐CT further characterized bone and ureteral metastatic lesions. Subsequently, the patient was given a CT‐guided pulmonary lesion biopsy. The pathology of lung mass indicated an atypical carcinoid NET, and immunohistochemistry (IHC) showed CK (+), CK7 (+), Vimentin (−), TTF‐1 (+), SyN (+), GATA‐3 (−), P63 (−), CK20 (−), Napsin A (several +), P40 (−), CD38 (−), Ki‐67 (20%–30%), Uroplakin II (−), CgA (+), CD56 (+), CT (−), CD117 (several +), TG (−), and INSM1 (+). Ultrasound‐guided breast lesion biopsy was consistent with a well‐differentiated metastatic NET, and IHC showed CK (+), CK7 (+), Vimentin (−), TTF‐1 (+), CD56 (+), SyN (+), CgA (+), GATA‐3 (−), P40 (−), Ki‐67 (15%), INSM1 (+). SSTR2 (−), and P53 (wild‐type). According to the pathological findings, we reviewed the previous examinations to comprehensively search the primary origin, but no primary tumor was found. The patient was diagnosed as a metastatic NET patient of unknown primary origin.

Given the multiple metastasis presentation and lack of an identifiable primary tumor, we proceeded with systemic therapy. To identify possible causes and potentially actionable mutations, a paired next‐generation sequencing (NGS)‐based genetic testing of 1021 cancer‐related genes (Geneplus‐Beijing Ltd., Beijing, China) was performed on both DNA extracted from the lung tumor tissue and leukocytes. Totally, seven somatic mutations were identified and shown in Table 1.

**TABLE 1:** Somatic mutations detected by next‐generation sequencing.

Single‐nucleotide variants				
Gene	Transcript	c.HGVS	p.HGVS	Allele frequency
*ARID2*	NM_152641.2	c.622G > A	p.G208R	51.2%
*PDILT*	NM_174924.1	c.1662G > T	p.K554N	36.2%
*INPP4B*	NM_001101669.1	c.1372G > T	p.V458L	36.0%
*RPTOR*	NM_020761.2	c.1357C > G	p.L453V	34.8%
*TOP2A*	NM_001067.3	c.2357C > A	p.P786H	34.7%
*HIST1H3G*	NM_003534.2	c.292G > A	p.E98K	23.7%

Fusions			
Gene	Transcript	Functional region	Allele frequency
*CEP44‐ALK*	NM_001145314.1; NM_004304.4	Exon9:Exon20	12.8%

Abbreviations: HGVS, Human Genome Variation Society.

**FIGURE 1:**
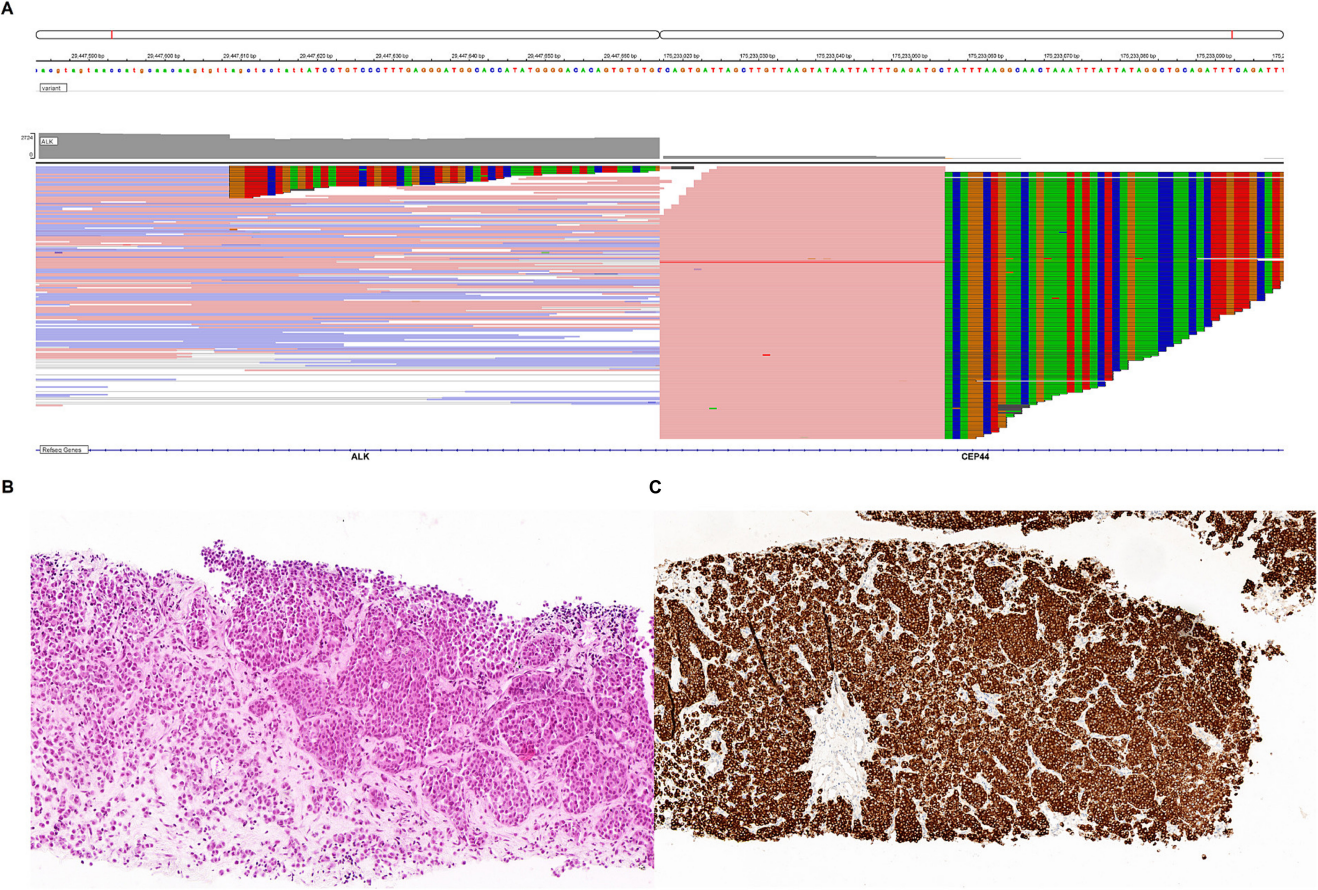
Identification of CEP44‐ALK rearrangement. (A) Next‐generation sequencing (NGS) identified a novel ALK fusion (CEP44‐ALK [exon9: exon20]) in the lung NET tissue. (B) Hematoxylin and eosin (H&E) staining of the neuroendocrine tumor. (C) Immunohistochemistry (IHC) result (Ventana‐D5F3 assay) further confirmed strong positivity of ALK protein expression in lung tumor tissue (× 10).

Of great interest, a novel ALK fusion (CEP44‐ALK [exon9: exon20]) was identified in tissue, with *CEP44* as the *ALK* fusion partner (Figure 1A). Hematoxylin and eosin (H&E) staining images of the puncture biopsy tissue reveal the NET components (Figure 1B). IHC result (Ventana‐D5F3 assay) of the lung tumor further confirmed a positive ALK protein expression (× 20) (Figure 1C).

**FIGURE 2:**
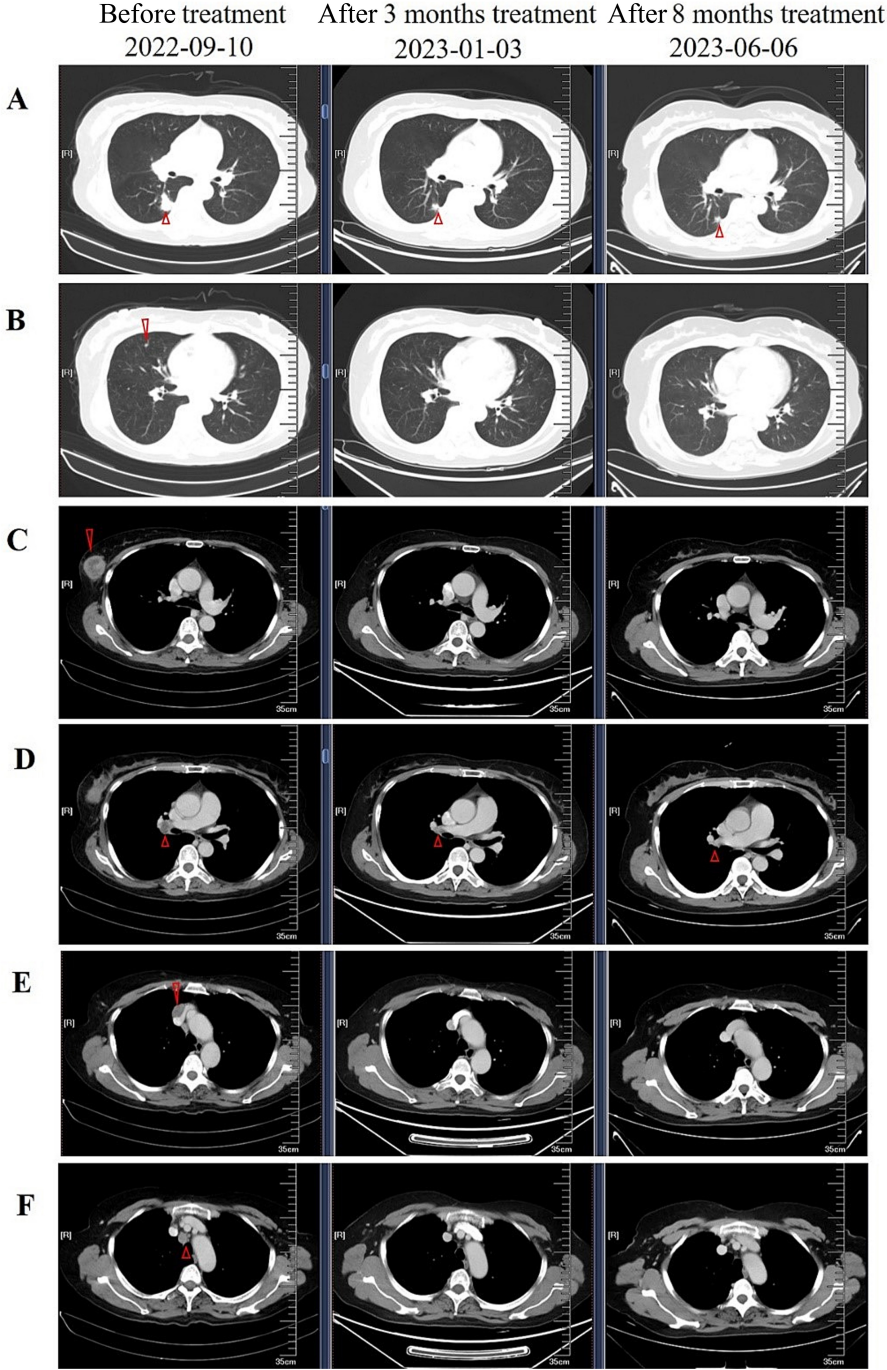
Dynamic imaging of the lesions at different stages of treatment. (A, B) Changes of the lesion in the lower lobe of the right lung and the small nodules in bilateral lung lobes after 3 months treatment with ensartinib in January 2023 and after 8 months treatment in June 2023, compared with the image before treatment in September 2022. (C) The lesion in the right breast disappeared after 3 months treatment with ensartinib. (D–F) The lymph nodes in the hilum and mediastinum of the right lung were reduced after 3 months treatment with ensartinib.

The patient did not receive any prior treatment. On October 7, 2022, treatment with ensartinib (225 mg/day) was initiated, along with bisphosphonate to control bone metastasis. After 3 months of ensartinib treatment, the CT scan showed the lesion in her lower lobe of the right lung shrank and the lesion in the right breast were disappeared. The small nodules in bilateral lung lobes and the lymph nodes in the hilum and mediastinum of the right lung were reduced (Figure 2). Based on the Response Evaluation Criteria In Solid Tumors v1.1, partial response (PR) was achieved. The patients underwent follow‐up dynamic CT examinations, and subsequent imaging revealed no significant evidence of recurrence. The patient received ensartinib treatment for 18 months until disease progression with the development of brain metastases in June 2024. Given the superior ability to cross the blood–brain barrier and its better control of brain metastases, lorlatinib was chosen as the second‐line therapy for this patient. After 1 month of lorlatinib treatment, the patient demonstrated a partial response (PR), with no significant adverse events reported. The patient is currently continuing oral treatment with lorlatinib.

## 3 Discussion

Patients with NET of unknown primary origin refractory to conventional therapy remain a huge clinical challenge that requires further interdisciplinary discussions to make comprehensive decisions [5].

NGS can provide abundant genetic information for NET patients and act as a genetic sentinel for treatment decision‐making, thus providing patients with more individualized therapeutic strategies. ALK rearrangement often serves as a driver mutation in cancers, inducing oncogenic hyperactivation of cytoplasmic tyrosine kinase activity irrespective of the fusion partners involved [2]. However, ALK rearrangement is exceedingly infrequent in NET, and most cases exhibiting ALK translocation are characterized by high‐grade histology and advanced disease stage, which strongly associates with an unfavorable prognosis. This is the first report of NET harboring a novel CEP44‐ALK rearrangement showing dramatic response to ensartinib. Moreover, we reviewed the previous literature demonstrating the use of ALK‐TKIs in NET (Table 2), including the fusion partners, ALK‐TKIs, and clinical outcomes.

**TABLE 2:** Clinical characteristics and therapeutic effect of ALK rearrangement in neuroendocrine tumor.

Fusion partner	Testing method	Pathology	ALK‐TKIs	Outcomes	Reference (DOI)
EML4	NGS	LCNET	Alectinib	More than 60 months survival	10.1016/j.cllc.2022.12.004
EML4	NGS or NGS/IHC	LCNETs	Alectinib	PFS 22 or 32 months	10.3389/fonc.2023.1227980
SMC5	NGS (ctDNA)	Atypical NET	Alectinib	60% shrinkage of brain lesion	10.1634/theoncologist.2017‐0054
PLB1	FISH/NGS/IHC	LCNET	Crizotinib	PFS 6 months	10.1016/j.cllc.2020.05.026
KIF5B	FISH/IHC	LCNET	Crizotinib/Alectinib	PFS 24 months	10.21873/anticanres.13127
NA	FISH	LCNET	Alectinib	PR	10.2169/internalmedicine.9368‐17
EML4/KLC1	FISH/NGS/IHC	LCNET	Crizotinib	PR	10.3346/jkms.2018.33.e123
EML4	NGS	LCNET	Alectinib	PR	10.21037/atm‐22‐6062
NA	FISH/NGS/IHC	LCNET	Alectinib	PR	10.1016/j.jtocrr.2023.100538
NA	IHC/FISH	LCNET	Alectinib	PR	10.3390/curroncol29020072

Abbreviations: FISH, fluorescence in situ hybridization; IHC, immunohistochemistry; LCNET, large cell neuroendocrine tumor; NGS, next‐generation sequencing; PFS, progression‐free‐survival; PR, partial response.

In our case review, we identified carcinogenic or targetable genetic alterations in ALK, including four cases of EML4‐ALK rearrangement, one case of SMC5‐ALK rearrangement, one case of KIF5B‐ALK rearrangement, one case of KLC1‐ALK rearrangement, and one case of PLB1‐ALK rearrangement. Due to the limitations of detection methods, specific details regarding mutation types were not available for the remaining three ALK mutation cases. Disease control rate (DCR) assessments were performed for all 10 patients, yielding a result of 100%. Three patients received crizotinib as first‐line therapy, with one patient subsequently treated with alectinib as second‐line therapy. Seven patients were treated with alectinib as first‐line therapy, with one patient achieving a 60% reduction in brain lesion size and another patient demonstrating a survival period of 60 months.

Several methods are available for detecting ALK mutations, each with its own strengths and weaknesses. Common techniques include fluorescent in situ hybridization (FISH), IHC, RT‐PCR, and NGS, primarily used on tissue samples. FISH is the gold standard for identifying ALK fusions in NSCLC, whereas the FDA‐approved Ventana ALK IHC (D5F3) aids in treatment decisions. However, both IHC and FISH rely heavily on pathologist interpretation and are susceptible to false positives due to signal instability and low sensitivity, which may result in missed treatment opportunities for some patients. In contrast, NGS can sequence millions of DNA fragments simultaneously, allowing for the cost‐effective detection of numerous tumor‐related genes without needing prior knowledge of specific mutations. This enhances the effectiveness of methods like IHC and FISH.

The previous studies suggested that ALK‐rearranged NETs represent a distinct molecular subtype characterized by their aggressive behavior [6]. Given the limitations of conventional treatment approaches, targeted therapy emerges as a potential avenue for therapeutic intervention in this specific patient subset, holding promise for improved clinical outcomes. Akhoundova et al. [6] demonstrated that the use of IHC to identify ALK rearrangement in NET was not as effective as in NSCLC due to a high prevalence of false‐positive cases and low specificity. False‐positive ALK expression also occurs in other types of tumors, such as neuroblastoma, which may be mediated by MYCN amplification [7]. However, in NSCLC, ALK positivity detected via IHC is commonly regarded as typical prompt of ALK‐TKI treatment, despite occasional instances of reported false positives and discordant results associated with ALK amplifications or activating mutations identified through FISH and NGS [8, 9]. In this study, they reported two metastatic lung NET patients with ALK rearrangement received rapid response to alectinib. Furthermore, Ghimire et al. [10] presented a high‐grade lung NET patient with ALK rearrangement exhibited a favorable response to alectinib. In the study by Wang et al. [11], an atypical NET with diffuse brain metastases and ALK rearrangement identified by ctDNA started on alectinib with rapid shrinkage of the disease and achieved a PR with resolution of brain metastatic lesion. Chen et al. [12] reported two metastatic lung NETs with EML4‐ALK rearrangement, which were treated with ALK‐TKIs and achieved a rapid therapeutic PR. Moreover, we summarized the documents about efficacy of ensartinib, as well as the available options following ensartinib resistance. Guo et al. [13] present a 71‐year‐old Chinese female with lung adenocarcinoma who developed acquired EML4‐ALK fusion after sequential treatment with erlotinib and osimertinib. Despite progression on osimertinib, third‐line ensartinib therapy led to significant tumor regression and normalization of serum markers within 1 month. The response to ensartinib lasted over 14 months, surpassing the effectiveness of alectinib and crizotinib in similar osimertinib‐resistant cases. Cheng et al. [14] describe a female patient with advanced NSCLC harboring an EML4‐ALK gene fusion who developed disease progression just 3 months after first‐line treatment with alectinib. Next‐generation sequencing identified two secondary mutations, including the novel E803Q mutation, which likely conferred resistance to second‐generation ALK‐TKIs like alectinib, ensartinib, and ceritinib, resulting in poor response and a survival of only 7 months. Song et al. [15] describe a 70‐year‐old patient with STRN‐ALK and TP53 mutations who was treated with ensartinib for advanced cancer. Despite developing COVID‐19, which led to heart failure and respiratory failure, the patient had a good clinical outcome and tolerated the treatment well, highlighting the potential for effective management even in older patients with comorbidities.

In our study, a metastatic NET with novel CEP44‐ALK rearrangement was described. The novel CEP44‐ALK protein synthesized by gene expression promoter and the full ALK kinase domain was confirmed to be expressed through an IHC assay, suggesting it may act as a “driver mutation” rather than a “passenger mutation” in this disease. Notably, the CEP44‐ALK rearrangement has not been reported in any other studies or databases, lacking the evidence for ALK‐TKI treatment. This case suggests that a NET patient with multiple metastases harboring the novel CEP44‐ALK rearrangement may exhibit promising efficacy to ensartinib.

## Author Contributions

HYC wrote the first draft and contributed with figure preparation and reference organization. YXW and XW contributed with a critical literature search and editing of the manuscript. QMW contributed with refinement of the study design, manuscript review, and clinical care. QXX designed the work, performed histopathologic diagnosis, took micrographs, and conducted a critical review of the initial and final drafts. All authors have read and agreed to the published version of the manuscript.

## Ethics Statement

Ethical review and approval were waived for this study due to it being a case report.

## Consent

Informed consent was obtained from all individual participants included in the study.

## Conflicts of Interest

Kai Wang is an employee of Geneplus‐Beijing. The other authors have no conflicts of interest to declare.

## Data Availability

Data sharing is not applicable to this article, as no datasets were generated or analyzed during the current study.

## References

[1] Q. Zheng , M. Zheng , Y. Jin , et al., “ALK‐Rearrangement Neuroendocrine Carcinoma of the Lung: A Comprehensive Study of a Rare Case Series and Review of Literature,” Oncotargets and Therapy 11 (2018): 4991–4998. 30154667 10.2147/OTT.S172124 PMC6103612

[2] Z. Wang , Y. Han , H. Tao , et al., “Molecular Characterization of Genomic Breakpoints of ALK Rearrangements in Non‐Small Cell Lung Cancer,” Molecular Oncology 17, no. 5 (2023): 765–778. 36423218 10.1002/1878-0261.13348 PMC10158786

[3] L. Horn , Z. Wang , G. Wu , et al., “Ensartinib vs Crizotinib for Patients With Anaplastic Lymphoma Kinase‐Positive Non‐Small Cell Lung Cancer: A Randomized Clinical Trial,” JAMA Oncology 7, no. 11 (2021): 1617–1625. 34473194 10.1001/jamaoncol.2021.3523 PMC8414368

[4] A. Leblanc , S. Owen , P. O. Fiset , A. L. Gomez Corrador , J. Isenberg , and N. Bouganim , “Metastatic Large‐Cell Neuroendocrine Lung Carcinoma With ALK Fusion Oncogene With Partial Response to Alectinib,” JCO Precision Oncology 5 (2021): 802–807. 34994612 10.1200/PO.20.00348

[5] K. Alexandraki , A. Angelousi , G. Boutzios , G. Kyriakopoulos , D. Rontogianni , and G. Kaltsas , “Management of Neuroendocrine Tumors of Unknown Primary,” Reviews in Endocrine & Metabolic Disorders 18, no. 4 (2017): 423–431. 29199361 10.1007/s11154-017-9437-9

[6] D. Akhoundova , M. Haberecker , R. Fritsch , et al., “Targeting ALK in Neuroendocrine Tumors of the Lung,” Frontiers in Oncology 12 (2022): 911294. 35756632 10.3389/fonc.2022.911294 PMC9214311

[7] M. K. Hasan , A. Nafady , A. Takatori , et al., “ALK Is a MYCN Target Gene and Regulates Cell Migration and Invasion in Neuroblastoma,” Scientific Reports 3 (2013): 3450. 24356251 10.1038/srep03450 PMC3868972

[8] M. I. Ilie , C. Bence , V. Hofman , et al., “Discrepancies Between FISH and Immunohistochemistry for Assessment of the ALK Status Are Associated With ALK ‘Borderline’‐Positive Rearrangements or a High Copy Number: A Potential Major Issue for Anti‐ALK Therapeutic Strategies,” Annals of Oncology 26, no. 1 (2015): 238–244. 25344360 10.1093/annonc/mdu484

[9] Y. Togashi , H. Mizuuchi , Y. Kobayashi , et al., “An Activating ALK Gene Mutation in ALK IHC‐Positive/FISH‐Negative Nonsmall‐Cell Lung Cancer,” Annals of Oncology 26, no. 8 (2015): 1800–1801. 26002608 10.1093/annonc/mdv240

[10] B. Ghimire , A. Pokharel , U. Karki , S. Thapa , and M. M. Chisti , “Anaplastic Lymphoma Kinase (ALK) Positive Neuroendocrine Tumor of Lung With Favorable Response to Alectinib (ALK Inhibitor),” Clinical Lung Cancer 24, no. 3 (2023): e113–e116. 36690569 10.1016/j.cllc.2022.12.004

[11] V. E. Wang , L. Young , S. Ali , et al., “A Case of Metastatic Atypical Neuroendocrine Tumor With ALK Translocation and Diffuse Brain Metastases,” The Oncologist 22, no. 7 (2017): 768–773. 28507205 10.1634/theoncologist.2017-0054 PMC5507651

[12] Q. Chen , J. Zhang , X. Wang , et al., “Two Case Reports: EML4‐ALK Rearrangement Large Cell Neuroendocrine Carcinoma and Literature Review,” Frontiers in Oncology 13 (2023): 1227980. 38023218 10.3389/fonc.2023.1227980 PMC10646488

[13] Y. Guo , R. Zhang , Y. Meng , L. Wang , L. Zheng , and J. You , “Case Report: Durable Response of Ensartinib Targeting EML4‐ALK Fusion in Osimertinib‐Resistant non‐small Cell Lung Cancer,” Frontiers in Pharmacology 15 (2024): 1359403. 39135785 10.3389/fphar.2024.1359403 PMC11317239

[14] X. Cheng , J. Liu , Q. Hu , Y. Gao , and L. Zhou , “A Novel Secondary ALK Gene Mutation Which Resistant to Second‐Generation TKIs: A Case Report and Literature Review,” Frontiers in Oncology 29, no. 14 (2024): 1430350. 10.3389/fonc.2024.1430350 PMC11390379 39267820

[15] G. Q. Song , Y. Z. Li , W. Kong , and G. Q. Hu , “Case Report: A Rare Case of non‐small Cell Lung Cancer With STRN‐ALK Fusion in a Patient in Very Poor Condition Treated With First‐Line Ensartinib,” Frontiers in Oncology 13 (2023): 1235679. 37810968 10.3389/fonc.2023.1235679 PMC10556511

